# Update on the INTEnsive ambulance-delivered blood pressure Reduction in hyper-ACute stroke Trial (INTERACT4): progress and baseline features in 2053 participants

**DOI:** 10.1186/s13063-023-07861-5

**Published:** 2023-12-20

**Authors:** Chen Chen, Yapeng Lin, Feifeng Liu, Xiaoying Chen, Laurent Billot, Qiang Li, Yiija Guo, Hueiming Liu, Lei Si, Menglu Ouyang, Chunfang Zhang, Hisatomi Arima, Philip M. Bath, Gary A. Ford, Thompson Robinson, Else Charlotte Sandset, Jeffrey L. Saver, Nikola Sprigg, H. Bart van der Worp, Gang Liu, Lili Song, Jie Yang, Gang Li, Craig S. Anderson, for the INTERACT4 Investigators

**Affiliations:** 1 Department of Neurology, Shanghai East Hospital, School of Medicine, Tongji University, 1800, Yuntai Road, Shanghai, 200120 China; 2 The George Institute for Global Health, Faculty of Medicine, UNSW, PO Box M201, Missenden Rd., Sydney, NSW 2050 Australia; 3 The George Institute for Global Health, Room 011, Unit 2, Tayuan Diplomatic Office Building, No. 14 Liangmahe Nan Lu, Chaoyang District, Beijing, 100600 China; 4 Department of Neurology, Clinical Medical College and The First Affiliated Hospital of Chengdu Medical College, Chengdu, China; 5 International Clinical Research Centre, Chengdu Medical College, Chengdu, China; 6 Beijing Municipal Key Laboratory of Clinical Epidemiology, School of Public Health, Capital Medical University, Beijing, China; 7 Beijing Chest Hospital, Capital Medical University, Beijing, China; 8 School of Health Sciences, Western Sydney University, Campbelltown, Australia; 9 Translational Health Research Institute, Western Sydney University, Penrith, Australia; 10 Shanghai Pudong New Area Medical Emergency Centre, Shanghai, China; 11 Department of Preventive Medicine and Public Health, Faculty of Medicine, Fukuoka University, Fukuoka, Japan; 12 Stroke Trials Unit, Mental Health & Clinical Neuroscience, University of Nottingham, Nottingham, UK; 13 Oxford University Hospitals NHS Foundation Trust and University of Oxford, Oxford, UK; 14 College of Life Sciences and NIHR Leicester Biomedical Research Centre, University of Leicester, Leicester, UK; 15 Department of Neurology, Oslo University Hospital, Oslo, Norway; 16 Norwegian Air Ambulance Foundation, Oslo, Norway; 17 University of California, Los Angeles, USA; 18 Department of Neurology and Neurosurgery, University Medical Centre Utrecht, Utrecht, the Netherlands; 19 Clinical Medical College and The First Affiliated Hospital of Chengdu Medical College, Chengdu, China; 20 Department of Neurology, Sichuan Academy of Medical Sciences & Sichuan Provincial People’s Hospital, University of Electronic Science and Technology of China, 32# W. Sec 2, 1st Ring Road, Chengdu, China; 21 Sichuan Provincial Key Laboratory for Human Disease Gene Study, Chengdu, China

## Abstract

**Background and aims:**

Uncertainty persists over the effects of blood pressure (BP) lowering in acute stroke. The INTEnsive ambulance-delivered blood pressure Reduction in hyper-Acute stroke Trial (INTERACT4) aims to determine efficacy and safety of hyperacute intensive BP lowering in patients with suspected acute stroke. Given concerns over the safety of this treatment in the pre-hospital setting, particularly in relation to patients with intracerebral hemorrhage, we provide an update on progress of the study and profile of participants to date.

**Methods:**

INTERACT4 is an ongoing multicentre, ambulance-delivered, randomized, open-label, blinded endpoint trial of pre-hospital BP lowering in patients with suspected acute stroke and elevated BP in China. Patients are randomized via a mobile phone digital system to intensive (target systolic BP [SBP] <140mmHg within 30 min) or guideline-recommended BP management. Primary outcome is an ordinal analysis of the full range of scores on the modified Rankin scale scores at 90 days.

**Results:**

Between March 2020 and April 2023, 2053 patients (mean age 70 years, female 39%) were recruited with a mean BP 178/98 mmHg in whom 45% have a diagnosis of primary intracerebral hemorrhage upon arrival at hospital. At the time of presentation to hospital, the mean SBP was 160 and 170mmHg in the intensive and control groups (Δ10 mmHg), respectively. The independent data and safety monitoring board has not identified any safety concerns and recommended continuation of the trial. The sample size was reduced from 3116 to 2320 after meetings in August 2022 as the stroke mimic rate was persistently lower than initially estimated (6% vs 30%). The study is expected to be completed in late 2023 and the results announced in May 2024.

**Conclusions:**

INTERACT4 is on track to provide reliable evidence of the effectiveness of ambulance-delivered intensive BP lowering in patients with suspected acute stroke.

**Trial registration:**

ClinicalTrials.gov NCT03790800; registered on 2 January 2019. Chinese Trial Registry ChCTR1900020534, registered on 7 January 2019.

## Introduction

Controversy persists over the balance of potential benefits and risks of intensive blood pressure (BP) lowering treatment in acute stroke [1, 2], but especially in the pre-hospital ambulance setting considering recent randomized evidence. The multicenter pre-hospital transdermal glyceryl trinitrate in patients with ultra-acute presumed stroke (RIGHT-2) trial conducted in the UK showed no overall effect of a transdermal glyceryl trinitrate patch compared to use of a sham patch in the intention-to-treat population [3]. However, the intervention resulted in greater bleeding, death, and disability, in the subgroup of patients with a final diagnosis of acute intracerebral hemorrhage [3]. Similar non-significant trends towards harm in patients with intracerebral hemorrhage that were noted by the data and safety monitoring board (DSMB) prompted the early termination of another clinical trial evaluating the use of transdermal glyceryl trinitrate in The Netherlands [4]. Most recently, the second intensive blood pressure control after endovascular thrombectomy for acute ischemic stroke trial (ENCHANTED2/MT) also underwent early termination because intensive BP lowering to a systolic target of <120mmHg (versus a target of <140mmHg) resulted in worse functional outcome in patients with persistent hypertension after successful endovascular clot retrieval for acute ischemic stroke from occlusion of a large intracranial vessel [5]. We, therefore, considered it appropriate to provide a progress report on the fourth INTEnsive ambulance-delivered blood pressure Reduction in hyper-ACute stroke Trial (INTERACT4), which aims to determine the efficacy and safety of pre-hospital initiated BP-lowering treatment in patients with suspected acute stroke in China.

### Design summary

**Fig. 1:**
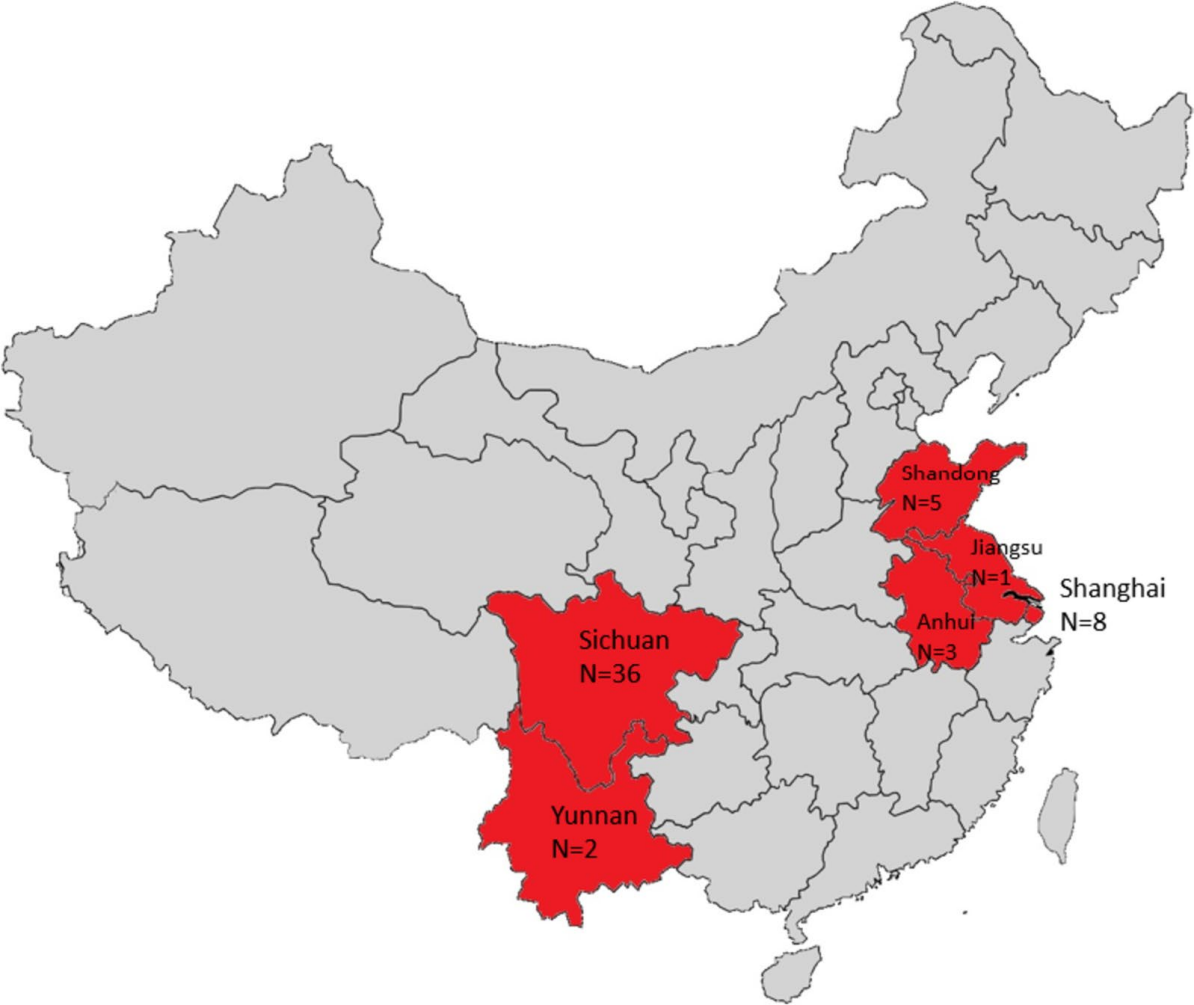
Regions of conduct of INTERACT4 in China. Includes numbers of participating hospitals in provinces

**Fig. 2:**
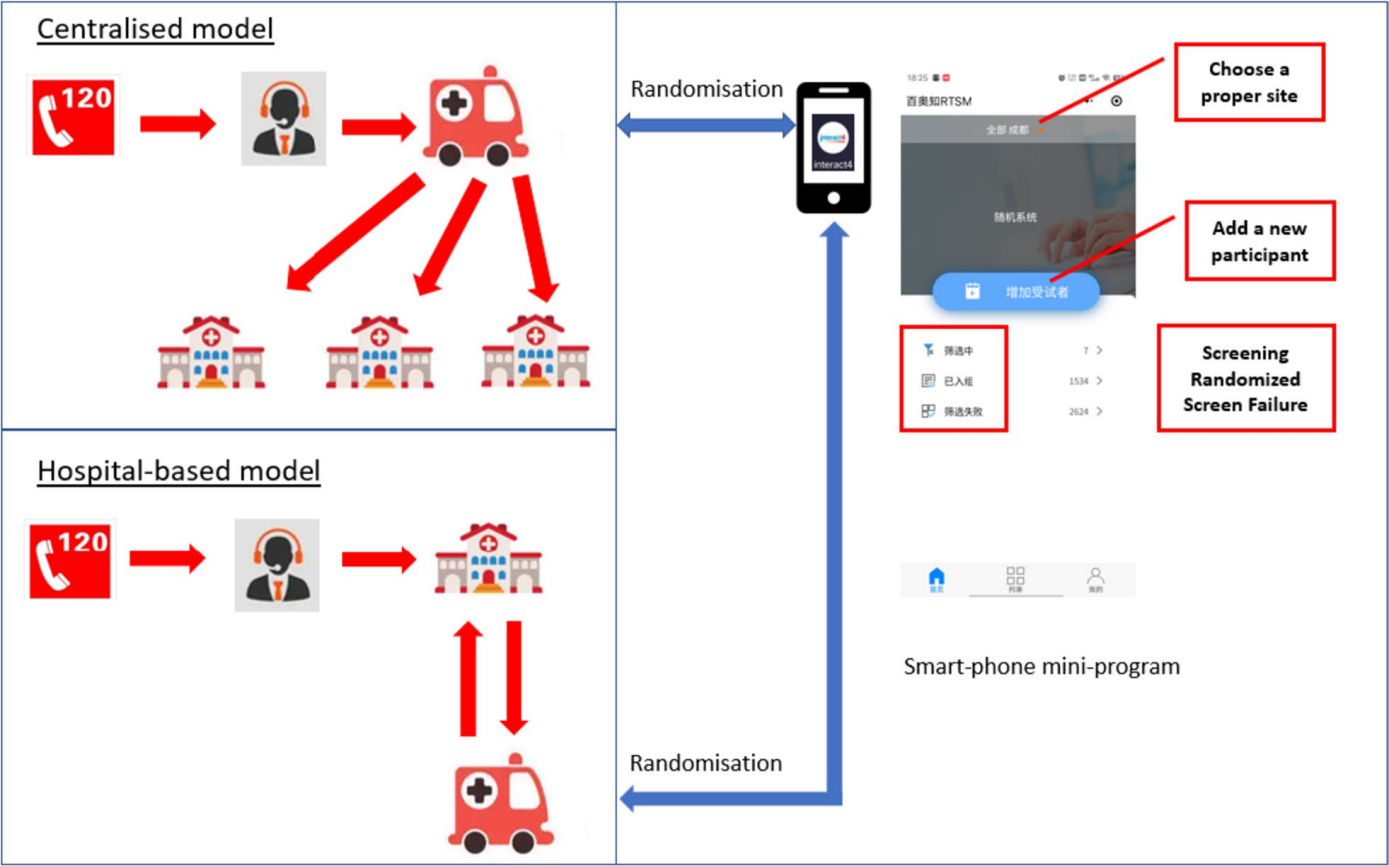
Ambulance systems and randomization process

INTERACT4 is a multicenter, ambulance-delivered, prospective, randomized, open-label, blinded endpoint (PROBE) assessed trial of pre-hospital BP lowering in suspected acute stroke patients with elevated systolic BP (SBP) ≥150mmHg undertaken in regions of China (Fig. 1), the details of which have been outlined [6]. In summary, screen-positive patients are randomized (1:1) by ambulance staff via smart-phone mini-program (Fig. 2) to intensive BP lowering group where an available fixed-dose bolus of intravenous urapidil or equivalent antihypertensive agent is administered as quickly as possible (target SBP 130–140mmHg within 30 min) or guideline-recommended BP lowering group (no BP lowering in the ambulance unless the SBP is very high [>220mmHg]). Both randomized groups of patients receive local guideline-recommended standard of care upon their arrival at hospital. The primary outcome is physical function according to an ordinal shift in the full range of scores on the modified Rankin scale (mRS) assessed at 90 days of follow-up by trained observers who are independent of the treating hospital teams.

### Recruitment and impact of COVID

**Fig. 3:**
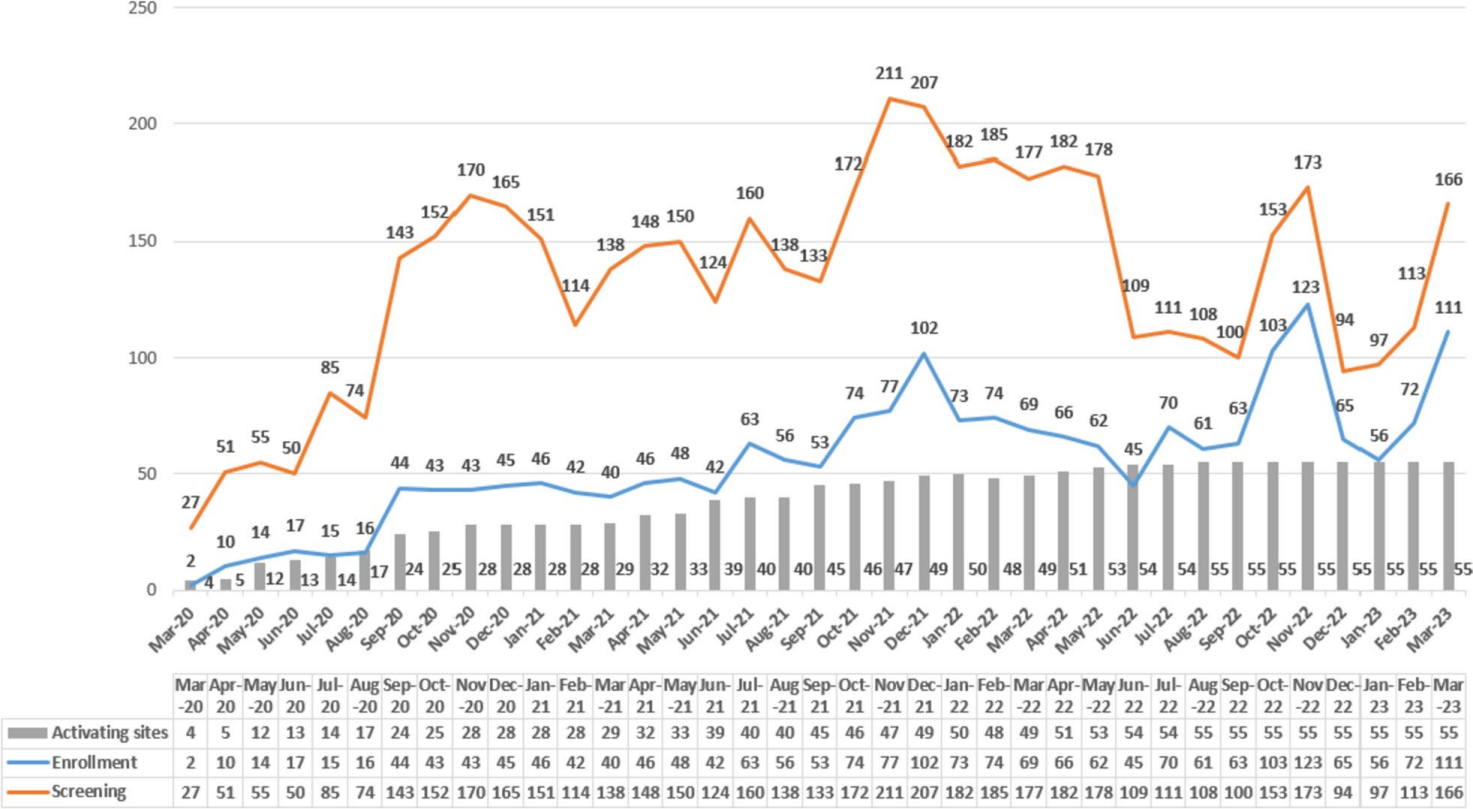
Recruitment by month (data extraction April 3 2023)

Patient enrolment commenced March 20, 2020, and increased slowly over the subsequent 6 months such that the recruitment increased to approximately 10 patients per month by December 2020 (Fig. 3). Recruitment further increased to 40–60 patients per month after 40 sites were activated by mid-2021, and peaked to 102 patients recruited in December 2021, the middle of winter when rates of major cardiovascular events are highest. Recruitment subsequently slowed down in the first half, and towards the end of 2022, in relation to the COVID outbreak which resulted in the redeployment of medical staff to testing centres and other activities, and members of the community avoiding calling ambulances and only attending hospitals with serious illnesses. Moreover, the research operations team faced travel restrictions due to COVID, resulting in delays in the entry of missing data, and a shift towards remote monitoring and online training of investigators. Recruitment subsequently picked up such that the required sample size is expected to be reached by late 2023. As of April 3, 2023, there are 55 hospital sites actively recruited patients from five provinces and Shanghai across China, with 4958 patients screened and 2053 (41.4%) patients enrolled. Table 1 shows that the main reasons for screen failure are due to being non-hypertensive (SBP <150mmHg) and having a delayed time (>2 h) from the onset of symptoms.

**Table 1:** Screen failure reasons

Reasons	*N*=4660^a^	%
SBP <150mmHg	1502	32
Time from the onset of symptoms >2 h	1361	29
FAST score of 1 or without arm deficiency	709	15
Deep coma	534	12
Severe comorbidity	239	5
Evidence of a seizure	106	2
Evidence of a head injury	92	2
Glucose <2.8mmol/L	85	2
Age <18 years	32	1

*FAST* Face, Arm, Speech, and Time scale, *SBP* Systolic blood pressure

^a^Reasons are not mutually exclusive

### Reduction in sample size

We had estimated that a sample size of 3116 would provide 90% power to detect a 22% reduction in the odds (common odds ratio of 0.78) of a worse functional outcome using ordinal logistic regression, on the assumption that 30% of patients would have a stroke mimic and 5% would have a missing mRS assessment. In October 2022, the Trial Steering Committee (TSC) decided to re-estimate the required sample size on the basis of a persistent reduction in the proportion of patients with a stroke mimic. From the blinded report generated for the fourth meeting of the DSMB in which an interim analysis was undertaken on September 14, 2022, the stroke mimic rate was 4–6%. The TSC reduced the required sample size to 2320; all other parameters remained the same. No safety concerns have been raised in DSMB reports. The updated Version 4.0 of the protocol with the modified sample size has been approved by all ethics committees.

### Reporting on the intervention (according to data extract April 3, 2023)

**Fig. 4:**
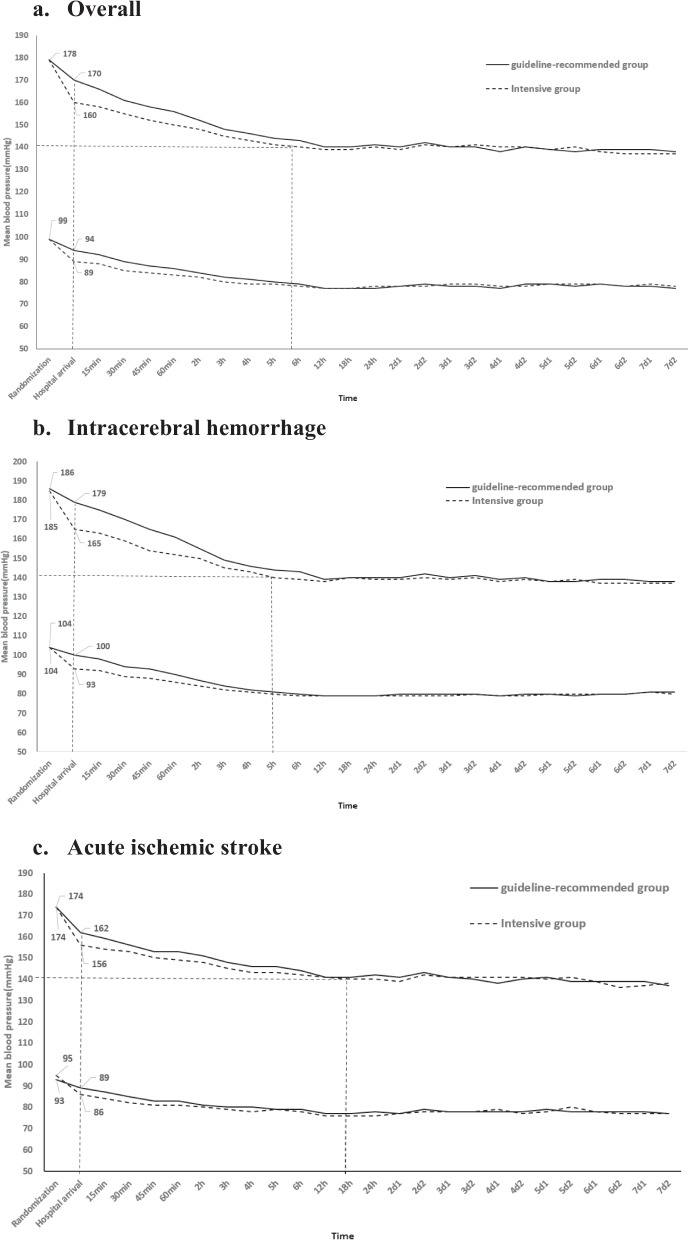
Mean systolic and diastolic blood pressure from randomization to day 7, overall and according to pathological subtype of stroke

The mean pre-randomization BP is 178/99 mmHg (Table 2). The magnitude of reduction in SBP from the time of randomization to the time of hospital arrival is 18 and 8mmHg in intensive group and control group, respectively, resulting in 10mmHg difference between groups, which gradually diminishes over the subsequent 7 days (Fig. 4a). Baseline SBP levels are higher and show a greater difference between randomized groups, for patients with acute intracerebral hemorrhage compared to patients with acute ischemic stroke (Fig. 4b, c).

**Table 2:** Characteristics of participants

Variable	*n*=2053
Time from onset to randomization, min	57 (38–83)
Time from onset to ambulance call, min	23 (10–47)
Time from onset to ambulance arrival, min	42 (25–69)
Age, years	70 (13)
Female	781/2017 (39)
Systolic BP, mmHg	178 (21)
Diastolic BP, mmHg	98 (16)
Heart rate, bpm	83 (16)
FAST score on assessment^1^	3 (2–4)
Glucose, mmol/L	7.3 (6.3–9.0)
NIHSS score at hospital arrival^2^	12 (6–18)
GCS score at hospital arrival^3^	13 (10–15)
Hypertension	1265/1777 (71.2)
Currently treated hypertension	887/1775 (50.0)
Coronary artery disease	210/1777 (11.8)
Other heart disease	99/1777 (5.6)
Atrial fibrillation	148/1777 (8.3)
Diabetes mellitus	302/1777 (17.0)
Hypercholesterolemia	30/1776 (1.7)
Current smoker	308/1773 (17.4)
Pre-stroke independent function (mRS score 0)	1140/1774 (64.3)
Anticoagulant use	47/1773 (2.7)
Aspirin/other antiplatelet agent	156/1771 (8.8)
Statin/other lipid lowering treatment	123/1770 (6.9)
Presumed pathology by day 7^4^	
Intracerebral hemorrhage	754/1676 (44.9)
Ischemic stroke	836/1676 (50.0)
Non-stroke	86/1676 (5.1)

Data are *n*/*N* (%), mean (SD), or median (IQR)

*FAST* Face, Arm, Speech, and Time scale, *GCS* Glasgow coma scale, *mRS* modified Rankin scale, *NIHSS* National Institutes of Health Stroke Scale, *BP* blood pressure

^1^Scores range from 0 to 4, 1 score for each Face/Arm/Speech/Time deficit

^2^Scores range from 0 to 42, with higher scores indicating greater neurological deficit

^3^Scores range from 15 (normal) to 3 (deep coma)

^4^Diagnosis according to the clinician’s interpretation of clinical features and results of brain imaging at the time of admission

### Characteristics of patients

Table 2 outlines the baseline characteristics of participants to date. Their mean age is 70.0 (12.6) years and 39% are female. The median time from onset of symptoms to randomization is 57 min, indicating that over half are commencing treatment within the “golden hour” of stroke rescue [7]. The median score on the National Institutes of Health stroke scale (NIHSS) at the time of hospital arrival is 12 (6–18), and for the Glasgow coma scale (GCS) is 13 (10–15), indicating that most have a moderate to severe neurological deficit. The frequencies of hypertension, diabetes mellitus, hypercholesterolemia, and coronary artery disease are 71.2, 17.0, 1.7, and 11.8%, respectively. There is near equal split of acute ischemic stroke and primary intracerebral hemorrhage. Figure 3 outlines the BP parameters overall and separately for those with acute ischemic stroke and intracerebral hemorrhage.

### Overview of the ambulance systems

The trial has two ambulance systems—centralized and hospital-based models—that represent the major medical care systems in China (Fig. 2). Table 3 outlines differences in their organization and implications for the research teams. The centralized model incorporates a standalone, independent emergency medical care organization, with its own staff of doctors, drivers, nurses, and stretcher bearers. This is linked to a series of ambulance subsites, with their size and location determined by the density and needs of a regional population. This model is flexible in its dispatch and delivery, with patients being delivered by ambulances dispatched from the nearest subsite to the nearest hospital, and is usually located in high-income areas where there is strong input and support from local government. This model is typical for the eastern regions of INTERACT4. Research teams were required to undertaken intensive levels and continuous training of investigators due to the high turnover of staff. Short delivery times meant that it was challenging to establish intensive BP lowering intervention in the ambulance. Although this model provided stable and continuous recruitment at eastern sites, the heavy workload of training and complexity of cooperation required between the ambulance system and hospitals, limited the ability to expand the network of other hospitals in the region.

**Table 3:** Differential characteristics of centralized and hospital-based ambulance systems

	Centralized model	Hospital-based model
**Characteristics**	Independent institute	Manage by hospital emergency department
	Independent workers	Well-experienced doctors and nurses from the hospital
	Flexible, short dispatch, and deliver time	Based on hospital resources, less input from government
	High employee turnover, need frequently training	Less flexible, more delivery time due to long distance
**Research issues**	Shorter time to intervention on ambulance	Adequate time for intervention on ambulance
	Stable recruitment	Easier to activate sites
	Great research training input	Stable and experienced doctors and nurses

The hospital-based model is organized around ambulances owned and operated by hospitals, usually those that are large and of a tertiary level. Experienced doctors and nurses of the emergency department of these hospitals are employed to work on the ambulance. This model has low levels of funding from local government and is more common in low-middle-income regions, such as across the western sites of INTERACT4. As only a few large hospitals have such an ambulance system, this model is less flexible and involves long dispatch and delivery times. This system allowed sufficient time to initiate and stabilize intensive BP management in ambulances. It was also more streamlined in terms of initiating and monitoring of sites, and in obtaining the necessary approvals and cooperation of various parties.

## Discussion

INTERACT4 is the largest ambulance trial in acute stroke, with over 2000 patients enrolled since April 2023 and many learnings gained. Recruitment has benefited from having doctors working in ambulances as part of routine practice in China. Their diagnostic and management skills were key reasons that allowed ethics committees to approve the use of a brief consent, or waiver of consent, according to circumstances for implementation of the intervention. It also explains why a low (6%) frequency of stroke mimics has been achieved when compared to figures (10–20%) reported in other trials in this setting [3, 4, 8, 9], and is the basis of our decision to reduce the sample size without compromising power and underlying assumptions required to determine the proposed treatment effect.

We purposefully included “hypertensive” FAST positive patients with a motor deficit in the screening criteria, primarily to increase the likelihood of including patients with a definite diagnosis of stroke. However, this approach has led to a high proportion of patients being included with severe neurological deficits, and in turn there are a higher proportion (45%) of cases of ICH where the background incidence is known to be high in China, compared to proportional frequencies of 16–23% reported in other ambulance studies in the west [3, 4, 8, 9]. INTERACT4 will, therefore, allow an assessment of the effects of ultra-early intensive BP management in patients with intracerebral hemorrhage, where clear time relations have not been well established to date [10–12].

The success of our trial to show an effect of early intensive BP lowering is heavily dependent on an early and satisfactory separation of BP being achieved between randomized groups, with interesting patterns emerging in the profile of SBP. To begin with, there were challenges in achieving a satisfactory BP reduction in the intervention group: investigators were cautious in their approach to this new treatment, as they were unfamiliar with assessing response and wished to avoid episodes of hypotension. With increasing experience, they became comfortable in providing an additional bolus of intravenous urapidil, the main intravenous antihypertensive treatment being used, and in controlling a rare event of symptomatic hypotension by the use of intravenous fluids and positioning the patient head-down with legs elevated. These aspects required repeated training of investigators to adapt to changing rosters and appointment of new staff. Training was also required in the transfer of care between staff in the ambulance and emergency department(s) to ensure there was continuity of treatment and maintenance of BP control. It is interesting that there are differential baseline SBP and response to treatment between those with acute ischemic stroke and intracerebral hemorrhage. As the proportion of patients with acute ischemic stroke due to large vessel occlusion is likely to be relatively high, it is reassuring that the DSMB has not identified any harms from early intensive BP lowering given findings of another trial of the intervention in this patient subgroup in China [5].

Finally, the trial has also been affected by the COVID pandemic, where reductions in the hospitalization of patients with acute stroke and the impact on research has been noted elsewhere [13, 14]. Site initiation and recruitment was initially slower than expected, and restrictions on travel meant that the research operational team undertook more remote than on-site visits for monitoring. In early 2022, a lockdown period in Shanghai changed the entire medical system with ambulances and participating hospitals prioritizing care for patients with COVID. Hospitals also experienced a shortage of resource, reductions in personnel, and a freeze on research activities. Continued participation of sites in other regions without lockdown minimized the impact of these activities on recruitment into the trial. Even so, there was still flow-on consequences to performance parameters, such as door-to-needle time, that may have impacted on management and outcomes for patients with acute stroke.

In summary, we have made satisfactory progress in the conduct of INTERACT4, with feasibility established and no harms noted in participants to date. After an interim analysis, protocol amendments were made in relation to reducing the sample size from 3116 to 2320 due to lower stroke mimic rate (6%) than expected (30%). The trial is on schedule for completion at the end of 2023 and for the results to be announced in mid-2024. In answering the question as to whether ambulance-delivered early intensive BP lowering in the hyperacute phase of stroke achieves benefits to patients, we anticipate high-quality evidence will be provided that we hope will impact for a change in clinical practice.

## Data Availability

Data sharing not applicable to this article as no datasets were generated or analyzed during the current study.
